# The Efficacy of Psycho‐Educational Interventions to Optimize Women's Sleep in Pregnancy: An Integrative Review

**DOI:** 10.1111/birt.12902

**Published:** 2025-03-10

**Authors:** Vishnuvarthini Visvanathan, Wendy Pollock, Yasmin Zisin, Suzanne Willey

**Affiliations:** ^1^ School of Nursing and Midwifery, Faculty of Medicine, Nursing, and Health Sciences Monash University Clayton Victoria Australia; ^2^ The Royal Women's Hospital Parkville Victoria Australia

**Keywords:** cognitive behavioral therapy, insomnia, pregnancy, psycho‐education, sleep disturbance, sleep hygiene

## Abstract

**Background:**

Poor sleep in pregnancy is associated with several adverse maternal and infant outcomes. Psychoeducational interventions may offer a safe and acceptable first‐line intervention to help with sleep disturbances.

**Aim:**

To identify and review studies that examined the effect of psycho‐educational interventions on sleep in pregnancy and to identify moderators in the treatment effects of the interventions.

**Methods:**

An integrative review methodology was used. A comprehensive search in five electronic databases retrieved 1250 articles. Eligible studies (*n* = 12) were assessed for methodological quality according to the “QualSyst” rapid appraisal tool. Data were extracted and recorded using a modified Covidence form. Quantitative data were summarized in a meta‐analysis or narrative synthesis. Qualitative data were narratively reported.

**Findings:**

Twelve studies with three different sleep interventions: Cognitive Behavioral Therapy for Insomnia (CBT‐I), Sleep Healthy Education (SHE) and relaxation training were included. Given the variation in study methodologies and interventions, only quantitative results from RCT trials using CBT‐I were summarized in the meta‐analysis. CBT‐I was found to be statistically significant in improving sleep quality in pregnancy (Standard Mean Difference = −0.78; 95% CI = −1.01, −0.54, *p <* 0.001). Few studies reported the efficacy of SHE and relaxation training. Potential moderators had no effect on the measured sleep quality outcome. Overall, psychoeducational interventions were acceptable to participants during pregnancy.

**Conclusion:**

There is insufficient evidence on which to base the recommendations about the effectiveness of all psychoeducational interventions to improve sleep. Based on the available literature, CBT‐I is an evidence‐based intervention to improve sleep quality in pregnancy.

## Graphical abstract

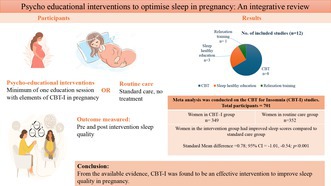

## 1 Introduction

Poor sleep in any form, duration, continuity, or quality has several short‐ and long‐term health consequences. For instance, in healthy adults, sleep disruption in the short term causes impaired emotional regulation, somatic pain, cognitive, memory, and performance deficits [1]. In the long term, poor sleep is related to hypertension, cardiovascular disease, and metabolic disturbances such as dyslipidemia, weight‐related issues, type‐2 diabetes mellitus, and colorectal cancer [1]. For childbearing women, pregnancy is a time of major biopsychosocial change and can be associated with increased sleep disturbances [2, 3, 4, 5]. Insomnia, defined as a sleep‐related concern where a person has trouble falling or staying asleep despite an adequate opportunity for sleep, is associated with significant impairment or distress in daytime function [6]. In pregnancy, the prevalence of insomnia is reported to increase with each trimester (first trimester: 44.2%; second trimester: 46.3%, and 63.7% in the third trimester), suggesting two out of three women suffer insomnia in late pregnancy [7, 8] with the potential to experience associated maternal and neonatal complications. For instance, sleep disturbance in pregnancy increases the risk of antenatal and postnatal depression among women [9, 10] gestational diabetes [4], Pregnancy‐Induced Hypertension (PIH) [5], Pre‐eclampsia (PE) [4, 5], Intra‐Uterine Growth Restriction (IUGR) [5], preterm labor and birth [4, 5], painful and long labor, and a higher risk of instrumental and caesarean birth [4, 5, 7]. Furthermore, a recent study found a possible causal relationship between genetically predicted insomnia and the risk of miscarriage, perinatal depression, and low birth weight [11].

During pregnancy, women are reluctant to use pharmacological medications to improve sleep quality due to concerns these may have adverse effects on the fetus [12]. Furthermore, pharmacotherapeutics are not advised given the potential teratogenic effects of some medications [13]. In the general population, psycho‐educational interventions to prevent and treat sleep disorders are considered a safe first‐line strategy [14, 15]. These interventions include combining education sessions with elements of Cognitive Behavioral Therapy for Insomnia (CBT‐I) to provide knowledge about the various facets of the sleep disturbances and their management [16]. Research into the use of psycho‐educational interventions in pregnancy and as a strategy to prevent and treat sleep disturbances is growing rapidly [17, 18, 19, 20]; however, these studies are varied in the type of content delivered, mode of delivery, i.e., online, or face‐to‐face, group or individual session; contact time with health professionals for training, and the length of time for follow‐up after the intervention. Besides, systematic reviews have focused on quantitative studies [17, 18, 19, 20] and there has been no systematic examination of women's perspectives on interventions or potential moderators.

The aim of this integrative review is to identify and review studies that examined the effect of psycho‐educational interventions on sleep in pregnancy and to identify moderators in the treatment effects of the interventions. Potential moderators include frequency of treatment, method of education delivery (e‐health vs. face‐to‐face or hybrid), treatment provider (psychologist, midwife, nurse, researcher), treatment goal (treat or prevent), baseline mental and physical health of the women, number of sessions, type of control, and risk of bias.

## 2 Methods

This integrative review is reported in accordance with the Preferred Reporting Items for Systematic reviews and Meta‐analyses (PRISMA) reporting guidelines [21]. A study protocol was developed by the authors, registered in PROSPERO (CRD42022384760) and used as a guide to conduct this review based on the following PICO search criteria.

### 2.1 Inclusion Criteria

#### 2.1.1 Population (P)

All pregnant women, regardless of age, gestation, or demographic details, were included. Women with and without self‐reported sleep disturbances or insomnia, as well as those with a formal diagnosis of sleep disorders in pregnancy were included to understand the effectiveness of the intervention in the prevention (i.e., universal intervention to prevent sleep disturbances) and treatment (i.e., manage existing sleep disturbances).

#### 2.1.2 Intervention (I)

Pregnant women need to have attended at least one session of psycho‐educational intervention focusing on maternal sleep in pregnancy. There was no restriction on the mode of delivery (face to face vs. online vs. hybrid), the treatment provider (psychologist, nurse midwife, researcher), treatment goal (treat or prevent), or number of sessions.

The interventions studied were psycho‐educational interventions aimed at improving sleep in pregnancy through at least one or more of the CBT–I strategies such as:

1. Cognitive interventions (e g., changing dysfunctional attitudes and beliefs towards sleep)
2. Behavioral interventions such as relaxation training, mindfulness strategies, and stimulus control (e g., minimizing electronic use in bed, only using bed for sleep)
3. Sleep restriction (e g., reducing bedtime to maintain homeostatic sleep pressure)
4. Sleep hygiene (behavioral and environmental recommendations intended to promote healthy sleep)
5. Educational intervention (information on connection between thoughts, feeling and behavior, and sleep).

#### 2.1.3 Comparison (C)

No treatment, standard care, wait‐list control, or treatment as usual, or other pharmacological and non‐pharmacological methods.

#### 2.1.4 Outcome (O)

The primary outcome was sleep‐related and measured pre‐and post‐intervention. Sleep‐related outcomes included sleep quality; degree of sleep disturbance; insomnia symptoms; and other sleep parameters (duration, quality, sleep efficiency index and wake after sleep onset, and sleep efficiency index); mood (depression, anxiety, positive affect, quality of life); fatigue; and somnolence.

#### 2.1.5 Study Types

Quantitative, qualitative, mixed method study designs, and individual primary studies previously cited in systematic reviews were included.

### 2.2 Exclusions and Limitations

For the purposes of the review, women in the postnatal period were not included as the sleep needs and interventions used for postnatal women are different and unique due to caring for a newborn and infant sleep patterns [17, 18]. Studies that examined the perinatal period were included only if the antenatal and postnatal findings were reported separately, and in these, only the pregnancy results were included in the review. The primary outcome of studies included is sleep quality. However, studies were not excluded if mood disorders were measured concurrently as a co‐primary outcome or as a secondary outcome.

Sources that were systematic reviews, theses, dissertations, gray literature, opinion pieces, letters, and editorials were excluded. As CBT emerged as a dominant psychotherapy modality since the work of Dr. Beck and colleagues in 1976 [22], the search was limited to publications after this year. Language was restricted to only English.

### 2.3 Information Sources and Search Strategy

Relevant studies were identified from the following databases: CINAHL, Embase, Ovid Medline, PsycINFO, and SCOPUS. A systematic search strategy was designed using subject ‘MeSH’ headings and key terms specific to each database in consultation with the University librarian. The keywords and subject headings focused on: “Pregnancy” OR “Prenatal” OR “antenatal” OR “Antepartum” AND “Psycho education” OR “Cognitive behavioral therapy” OR “CBT” OR “Sleep hygiene” OR “Sleep health” AND “Sleep quality” OR “Insomnia”. The final search was conducted on the December 20, 2022 with full search strategies contained in Appendix S1.

### 2.4 Study Selection

Covidence systematic review software [23] was used for the initial screening, data extraction, and quality appraisal. Two authors (V.V. and Y.Z.) independently conducted the screening process based on the study inclusion and exclusion criteria. Any conflict that arose was resolved through discussion with the other two authors (S.W. and W.P.).

### 2.5 Data Extraction and Quality Assessment

A modified Covidence data extraction form [23] was used to extract data from the eligible studies. Furthermore, these eligible studies were assessed for the methodological quality and risk of bias using the “QualSyst” mixed‐method appraisal tool [24]. Two authors (V.V. and Y.Z.) conducted the data extraction and quality assessment independently. Disagreements were resolved through consensus among all authors.

### 2.6 Data Synthesis

Integrative review methodology with a narrative synthesis enabled the combination of a wide variety of literature on psycho‐educational sleep interventions [25]. Quantitative data were summarized statistically in meta‐analysis using the Review manager software [26] if the data available were sufficiently homogenous. The degree of heterogeneity was measured using the ${\mathrm{Chi}}^{2}$ ($X^{2}$) test. Fixed‐effect model was used to calculate the summary effect size only if *p* ≥ 0.1 and $I^{2}$ < 50%; otherwise, a random‐effect model was used [20, 27]. Standard Mean difference was used as varied sleep measurement tools were used to measure the effect sizes. Data were pooled using the 95% confidence intervals (CI). Studies which were varied and not included in the meta‐analysis were narratively synthesized. No qualitative studies were retrieved in the search. However, eight studies included in this review had qualitative findings that have been reported narratively [28, 29, 30, 31, 32, 33, 34, 35].

## 3 Results

### 3.1 Study Selection and Characteristics

**FIGURE 1:**
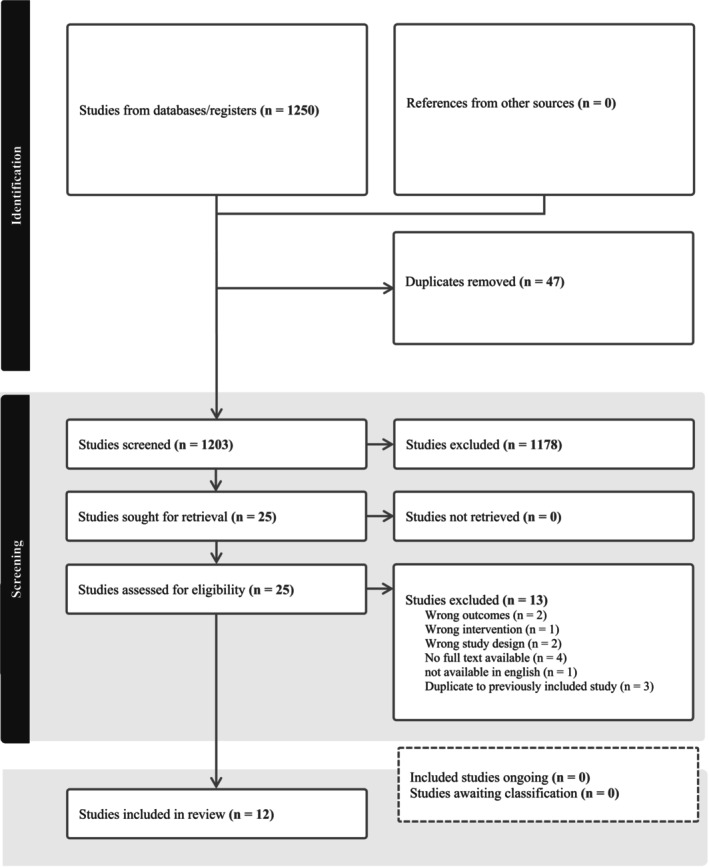
Prisma flowsheet. [Colour figure can be viewed at wileyonlinelibrary.com]

A total of 1250 articles were retrieved from the database search. Forty‐seven duplicates were removed, and 1203 articles were included for title and abstract screening. Following screening, 25 articles were eligible for the full text review. Further review for relevancy generated 12 articles for inclusion in this review. A PRISMA flow diagram is presented to summarize the screening (see Figure 1).

The 12 studies were conducted in the United States (*n* = 5) [29, 30, 31, 32, 35], Iran (*n* = 3) [34, 36, 37], Australia (*n* = 1) [28], Canada (*n* = 1) [33], New Zealand (*n* = 1) [38] and Turkey (*n* = 1) [39] and compared the effects and acceptability of a range of psycho‐educational sleep interventions. Of the retrieved studies, eight studies involved CBT‐I [28, 29, 30, 31, 32, 33, 35, 36]; three included Sleep Healthy Education (SHE) [34, 37, 38] and one studied relaxation training in pregnancy [39]. The search did not generate any single‐method qualitative studies. Qualitative data was collected using the self‐reported sleep quality measures [28, 29, 30, 31, 35] and participant feedback using open‐ended questions following the intervention [32, 33]. See Table 1 for the characteristics of included studies. A list of measured outcomes is included in Table S1.

**TABLE 1:** Characteristics of included studies.

Study ID	Country	Study design	Population description	Gestation	Sample	Cases	Control	Treatment goal	Sleep assessment tool
Bei 2021	Australia	RCT	Primiparous pregnant women without sleep disorders	30 weeks	163	81	82	Prevent	ISI & PROMIS Sleep disturbance
Cain 2020	United States	RCT	Obese or Overweight pregnant women with sleep disturbances	13–20 weeks	53	27	26	Treat	PSQI & ISI
Felder 2020	United States	RCT	Pregnant women with high insomnia risk	up to 28 weeks	208	105	103	Treat	ISI, PSQI, Sleep diary, GAD, EPDS
Hassanpour 2014	Iran	RCT	Pregnant women with sleep disturbances	26–32 weeks	150	73	77	Treat	PSQI
Kalmbach 2020	United States	RCT	Pregnant women without sleep disorders	25–30 weeks	91	46	45	Prevent	PSQI, ISI, PSAS
Khatibi 2021	Iran	RCT	Primiparous women without sleep disorders	12–24 weeks	56	28	28	Prevent	PSQI, DASS
Ladyman 2020	New Zealand	Longitudinal Pilot study	Primiparous women without sleep disorders	< 14 weeks	91	15	76	Prevent	General sleep disturbance scale, Epworth sleepiness scale, EPDS
Lee 2016	United States	Quasi‐experimental study	Primiparous women without sleep disorders	29–31 weeks	149	25	Group A:76 Group B:48	Prevent	Wrist actigraphy
Manber 2019	United States	RCT	Pregnant women with insomnia	18–32 weeks	179	89	90	Treat	ISI, EPDS
Ozkan 2018	Turkey	RCT	Primiparous pregnant women without sleep disorders	28–34 weeks	84	46	46	Prevent	PSQI
Rezaei 2014	Iran	RCT	Pregnant women with sleep disorders	16–20 weeks	112	56	56	Treat	PSQI, WHOQOL‐BREF questionnaire
Tomfohr‐Madsen 2017	Canada	Pilot study	Pregnant women with insomnia	12–28 weeks	13	13	0	Treat	PSQI, ISI, actigraphy and sleep diary, EPDS, PAS, MFSI‐SF

Abbreviations: DASS‐21, Depression, Anxiety and Stress Scale; EPDS, Edinburgh postnatal depression scale; GAD, General Anxiety Disorder Scale; ISI, Insomnia Severity Index; MFSI‐SF, Multidimensional Fatigue symptom severity‐short form; PAS, Pregnancy specific anxiety measures; PROMIS, Patient‐Reported Outcomes Information System; PSAS, Presleep Arousal Scale; PSQI, Pittsburgh Sleep Quality Index; WHOQOL‐BREF, World Health Organization Quality of Life Brief version.

### 3.2 Synthesis of Results

All 12 studies were included in the data synthesis. Nine studies were RCTs, two were pilot studies with pre‐post interventions, and one was a non‐randomized quasi‐experimental study. See Table 2 for a summary of study interventions and key findings.

**TABLE 2:** Study interventions and findings.

Study ID	Intervention	Intervention description	Intervention provider, language	Duration/follow up period	Comparison/study context	Findings
Bei 2021	CBT‐ based sleep intervention	CBT based sleep intervention A 50‐min standardized telephone session is delivered by a trained psychologist A series of emails containing text, graphics, and/or audio‐based intervention components Mothers who have difficulty applying the strategies could request brief email or telephone clarification from the psychologist	Psychologist English	In pregnancy period, intervention was delivered at 30 weeks (T1) Post intervention assessment: pregnancy endpoint 35 weeks (T2). Post partum follow ups included at 6 different timeframes	Healthy diet in pregnancy education given by a dietitian delivered using the same method (i.e., same timing, frequency, quantity of written information and staff contact)	The study found that compared to control, receiving CBT was associated with lower insomnia severity and sleep disturbance (two primary outcomes), and lower sleep‐related impairment at the pregnancy endpoint (*p* < 0.001)
Cain 2020	Group prenatal care with behavioral sleep intervention	7 group prenatal visits from 14 to 16 weeks (T1) and continued to term Participants randomized to the intervention received additional training in sleep hygiene adapted from CBT‐I online program Go! to Sleep. These sessions included stimulus control, mindfulness training, relaxation techniques and education in sleep hygiene. The content of the online modules was also covered in the group sessions Access to the Go! to Sleep online course was also given to participants	Group leaders were specialists in obstetrics and gynecology English	Post intervention assessment: Late third trimester, 36–41 weeks gestation (T2) and at 6–8 weeks postpartum (T3)	Group prenatal care with no information on the sleep hygiene	Findings noted an improvement in moderate to severe insomnia symptoms among the intervention group compared to the standard treatment in the third trimester (19.3 (± 6.0) vs. 14.7 (± 6.6) *p* < 0.05) that continued into the postpartum period The total PSQI scores did not demonstrate significant improvements but did demonstrate a moderate effect size
Felder 2020	Digital CBT‐I	Digital CBT‐I was delivered through Six weekly sessions The treatment content was based on CBT‐I manuals and included 5 main components: sleep restriction, stimulus control, cognitive therapy, relaxation techniques, and sleep hygiene and education Participants had access to a moderated online community and a library of sleep information	Sleepio (Big Health) Animated digital therapist English	six weekly sessions Post‐intervention assessment: at 10 weeks (postintervention) and 18 weeks (follow‐up) after randomization	Standard treatment	Women in the digital CBT‐I group had greater reductions in their insomnia symptom severity scores than women in the standard treatment group, 95% CI, difference = −0.36; 95% CI, −0.48 to −0.23); the difference between these rates was statistically significant (*p <* 0.001). Also, the depressive symptom severity in the treatment group reduced—0.22 vs. −0.01 and also the anxiety severity scores significantly reduced −0.19 vs. −0.002 in the CBT‐I group compared to the control group
Hassanpour 2014	Sleep health training	Two 2‐h sessions with a 1‐week interval, the intervention group received lecture‐based sleep health training in classes of 5 to 10. A training package along with a CD containing calming music	No information on the provider of the group lecture CD training package Language: not mentioned	Four weeks post intervention	Standard prenatal care	Results revealed that sleep health training improved the quality of sleep during pregnancy. The mean score [standard deviation] after the intervention for intervention and control groups were 4.95 [2.05] and 7.03 [0.78] respectively. The statistical analysis showed a significant difference at a 95% confidence level (*p* = 0.00)
Kalmbach 2020	Digital CBT‐ I	Six‐sessions of Digital CBT‐I Sleepio program. The intervention covered behavioral components (sleep restriction, stimulus control), cognitive components (e.g., cognitive restructuring, paradoxical intention), progressive muscle relaxation, and sleep hygiene. Only one modification was made to accommodate the pregnant women: in sleep restriction, time in bed could not be prescribed as < 6 h	Sessions were directed by an animated virtual therapist English	Six sessions Post intervention assessment: 1 week after completing treatment or upon discontinuing treatment (indicated by informing study personnel of their discontinuation or after 2 weeks of not engaging in treatment)	Online sleep education condition received six weekly emails based on the National Institutes of Health guide to healthy sleep.	From pre to posttreatment, CBT‐I patients reported reductions in Insomnia severity (*p <* 0.001), improved sleep quality in PSQI scale (*p <* 0.001) and increase in nightly sleep duration by 32 min (*p =* 0.008). No prenatal effect on the depression scores were observed
Khatibi 2021	Cognitive behavioral counseling	Intervention was conducted in five 90‐min weekly sessions at birth preparation classes 1st session—Introduction and Orientation to the CBT‐I 2nd session—Treatment rationale, sleep training, behavioral therapy, and bedtime guidelines and recommendations 3rd session—correcting/modifying cognitive behavioral errors, guiding the expectations of women while adhering to sleep hygiene education and treatment 4th session—adherence to treatment, expressing cognitive rationality, presenting teaching techniques of constructive anxiety, teaching the application of recording thoughts and how to complete constructive anxiety, thought recording, and resistance control page for women 5th session—adherence to treatment, removing any problem on behavioral component and cognitive component	Five 90‐min weekly sessions at birth preparation classes located in the comprehensive urban healthcare centres in Zanjan city Language: not mentioned	Post intervention assessment—immediately after last session (post‐test) and 8 weeks follow up	Routine prenatal care	The total score of the sleep quality showed significant improvement in the intervention group compared to the control group at follow‐up phases after controlling the baseline scores. This difference was significant (*p <* 0.001). Measures of depression, anxiety and stress levels using the DASS‐21 tool showed a significant improvement in the depression (Intervention 2.32 ± 2.68 vs. Control 6.56 ± 5.84; *p <* 0.05) and stress levels (Intervention 7.92 ± 5.55 vs. Control 11.92 ± 8.93; *p <* 0.05). Whilst the anxiety level was improved, the result was not statistically significant (*p =* 0.18)
Ladyman 2020	Sleep education	Three—trimester specific sleep education sessions Sessions were approximately 1–1.5 h in trimester 1, and 45–60 min in trimesters 2 and 3. The sleep education material covered three main sleep topics: General sleep and circadian information how and why sleep changes in each trimester trimester‐specific sleep support strategies The education sessions in each trimester were scheduled at times and places convenient to the women and were one‐on‐one and face‐to‐face	Not mentioned	Self‐reported depressive symptoms and five dimensions of self‐reported sleep data collected at 36 weeks gestation and 12 weeks postnatal were compared between the two groups	No sleep education	The findings support well‐evidenced reciprocal relationships between sleep and depressive symptoms. In addition to having significantly fewer depressive symptoms (*p =* 0.019), the intervention group also experienced significantly better sleep quality, continuity and sleep initiation in late pregnancy compared to the comparison group, although only sleep initiation (latency) remained significant after controlling for covariates. The anxiety subscale showed no difference between the groups (*p* = 0.18).
Lee 2016	Home‐based Sleep Enhancement Training	Four‐week Sleep Enhancement Training Pregnancy intervention was based on general principles of CBT‐I. Included components consist of cognitive re‐structuring, sleep hygiene, stimulus control, and relaxation training. Participants were instructed to listen to the auditory program in bed before going to sleep each night 1st week—15 min, Subsequent weeks—45 min (or until the participant fell asleep)	Pregnancy guidebook with weekly readings and auditory programs provided on an MP3player English	Post intervention assessments: At 36 weeks of gestation & 2 months postpartum	Participants in the 2 control groups were provided with a pamphlet containing recommendations for healthy eating and information about how diet can influence sleep	The intervention group had significantly longer sleep duration and less sleep disruption than both control groups. Intervention participants slept a mean total sleep time of 430 (95% CI 397–464) min during pregnancy compared with 420 (95% CI 403–438) and 417 (95% CI 395–439) min for the two control groups (*p* < 0.05)
Manber 2019	CBT‐I	Five individual therapy sessions The treatment protocol included general education about sleep and sleep during pregnancy, as well as information about healthy sleep habits; sleep restriction therapy, modified for pregnancy with initial time in bed recommendations equal to average total sleep time plus 30 min (and never less than 5.5 h); stimulus control; strategies for reducing cognitive and somatic hyperarousal; and relapse prevention. Cognitive therapy was provided through‐out the intervention to address sleep interfering thoughts as needed. Therapy also included education about infant sleep development and elements from Tips for Improving Postpartum Sleep	Trained therapists In English/Spanish	Five individual therapy sessions Assessment: Throughout therapy period up to 1 week after the therapy	Modified pseudo‐desensitization therapy for insomnia	Women assigned to CBT‐I experienced significantly greater reductions in insomnia severity and faster remission of insomnia disorder, with 64% of participants receiving cognitive behavioral therapy for insomnia experiencing remission of insomnia at the last available observation. Also a significant (*p <* 0.001) overall reduction in EPDS scores were observed (pre 7.7 ± 3.7; post 3.9 ± 3.6), however the effect size was small (Cohen's *d* = 0.15)
Ozkan 2018	Relaxation exercises	Four‐ week program consisting of 5 courses Relaxation exercises were given by using a handbook including relaxation exercises (information about how to perform relaxation, respiration control, and progressive relaxation exercises) and a relaxation exercises CD Relaxation exercises CD was composed of three parts apart from the introduction; it was prepared by the Turkish Psychologists Association based on E. Jacobson's relaxation techniques 4‐min: Introduction 10‐min: First part deep relaxation exercises as well as the points to take into consideration during the exercise. 30‐min: Second part introduces relaxation exercises with an instruction 30‐min: Third part includes only relaxation music without instructions. Women were asked to listen to the first and second parts of the CD together throughout the 4‐week program	Instructor + CD Language: not mentioned	Four‐ week program consisting of 5 courses Post intervention assessment—immediately after completion of 5 courses	Standard care	It was determined that relaxation exercises improved some sleep quality subscales including subjective sleep quality, sleep latency, sleep duration and habitual sleep efficiency, sleep disturbances, daytime dysfunction, and global sleep quality. The difference between the two groups was found to be statistically significant (*p <* 0.05)
Rezaei 2014	Sleep education	Four 1‐h weekly session of sleep education. 1st week—Characteristics of natural sleep, sleep changes in pregnancy and their causes 2nd week—Education of modification of most complaints concerning physical problems disturbing sleep in pregnancy 3rd week—Strategies to follow healthy sleep habits 4th week—Education on diet control, alcohol consumption, smoking and playing sports	Researcher Language: not mentioned	Four 1‐h sessions. Post intervention assessment—At 1 month and 2 months after the educational intervention	Standard care	Significant difference in sleep quality was seen in the intervention group after the intervention compared to before the intervention (At 1 month: 7.30 ± 3.33 vs. 8.76 ± 2.33, *p =* 0.009; 2 months: 6.88 ± 3.06 vs. 8.21 ± 2.85, *p* < 0.05). Mean scores of the quality of life showed an increase at 1 month (*p <* 0.000) and 2 months (*p <* 0.001) after intervention in the study group compared to the control group
Tomfohr‐Madsen 2017	CBT‐I	Five weekly 90‐min group sessions of CBT‐I. 1st session—Orientation to the CBT‐I group (e.g., use of daily sleep diaries) and set individualized treatment goals. 2nd session—Psycho‐education about sleep and sleep consolidation 3rd session—stimulus control 4th session—cognitive strategies to examine attitudes and beliefs about sleep 5th session—problem‐solving and relapse prevention. Participants received a manual containing readings summarizing the material being taught in‐session	PhD‐level clinical psychologist and a clinical psychology doctoral trainee, both trained in the delivery of group‐based CBT‐I English	Five weeks Post intervention assessments occurred an average of 9.08 (SD = 4.87) days after the completion of the final group session	No comparison	Significant reductions in insomnia symptoms and increases in subjective sleep quality Diary and actigraphy assessments of sleep also changed, such that participants reported less time in bed, shorter sleep onset latency (SOL), increased sleep efficiency (SE), and increased subjective total sleep time. Additionally, Pre and post treatment EPDS scoring to measure depression (9.54 vs. 6), Pregnancy specific anxiety measures (10.31 vs. 8.54) and the MFSI‐SF fatigue scores (32.00 vs. 3.38) all decreased reporting significant improvement (*p* < 0.05)in the participant's mood Effect sizes: medium to large

### 3.3 Methodological Quality and Risk of Bias

**FIGURE 2:**
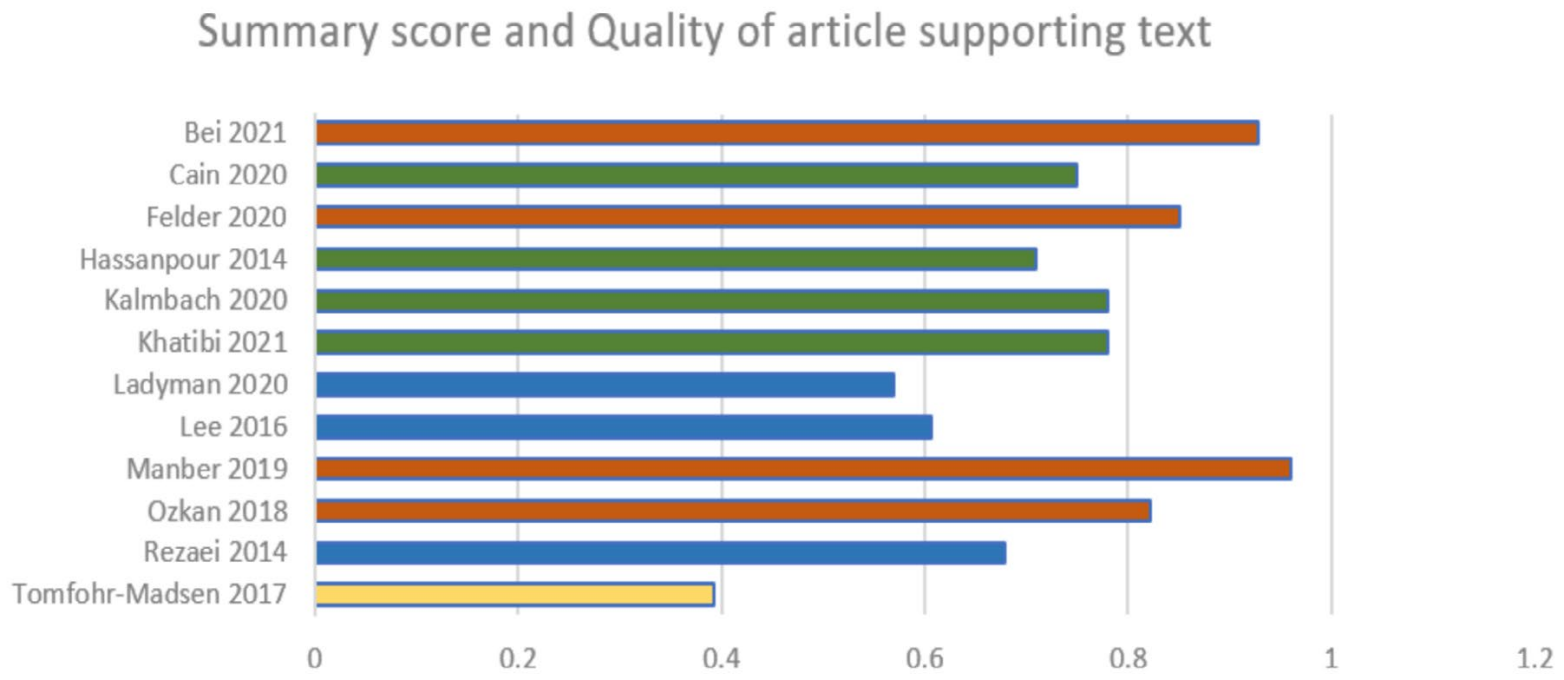
Quality Assessment of included studies. Red: Strong evidence (Summary score > 0.80); Green: Good evidence (score: 0.71–0.79); Blue: Adequate evidence (adequate score 0.50–0.70Yellow: Limited evidence (Score < 0.50). [Colour figure can be viewed at wileyonlinelibrary.com]

All nine RCTs conducted randomization of the sample with varied randomization strategies. Of the included RCTs, seven did not report on the assessors being blinded to the group intervention allocation [28, 29, 34, 35, 36, 37, 39]. One RCT reported a small sample size, which underpowered the study to detect the effect of the intervention [29]. Recruitment was conducted through convenience sampling [29, 34, 37, 39], attendance at child birthing classes [32], or via mail outs, phone calls, and flyer advertisements [28, 30, 35, 36, 38]. According to the ‘QualSyst’ rapid appraisal tool, most studies had good to strong scores of quality (See Figure 2). A detailed assessment is available in Table S2.

## 4 Effects of the Psycho‐Educational Interventions

### 4.1 Cognitive Behavioral Therapy for Insomnia

**FIGURE 3:**
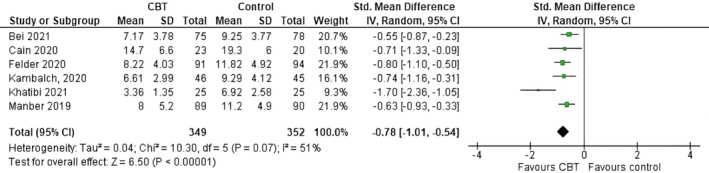
Forest plot of sleep assessment tools (PSQI & ISI) CBT‐I vs. control group. [Colour figure can be viewed at wileyonlinelibrary.com]

Six RCTs assessed CBT‐I [28, 29, 30, 31, 35, 36]. In total, 349 pregnant women were allocated to the CBT‐I intervention group, and 352 pregnant women to the control group (standard prenatal care, group care or routine health education). A range of validated sleep assessment tools were used, the most common were the Pittsburgh Sleep Quality Index (PSQI) [29, 30, 36] and the Insomnia Severity Index (ISI) [28, 29, 30, 31, 35]. Both PSQI and ISI assess for similar effect, that is, the extent of sleep disturbance (a lower score equates to better sleep quality). Meta‐analysis of these studies was conducted using Review Manager Software [26]. The summary effect size was calculated using a random effect model as the degree of heterogeneity was substantial ($I^{2}$= 51%). Standardized mean difference was used instead of the mean difference as the tools used to measure the effect varied between the studies. Data were pooled using 95% confidence intervals (CI). The sleep scores of the pregnant women in the CBT‐I intervention group was lower than that of the control group and the findings were statistically significant (Standardized mean difference = −0.78; 95% CI = −1.01, −0.54, *p <* 0.001) (see Figure 3).

One pre‐post CBT‐I intervention showed a significant difference in the PSQI (Mean change = 6.15, *p <* 0.001) and ISI (Mean change = 12.46, *p <* 0.001) scores in the intervention group compared to the control group [33]. A quasi‐experimental study by Lee et al. [32] on home‐based CBT‐I sleep intervention had a mean total sleep time of 430 min (95% CI 397–464), which was 10 and 14 min longer than control groups; however, the effect sizes for these group comparisons (d50.10 and 0.13, respectively) indicated minimal intervention effects during the pregnancy post‐intervention assessment [32].

### 4.2 Sensitivity Analysis

As only a few studies were appropriate for meta‐analysis, the random effects model may provide an inaccurate measure of the study variance [27]. Therefore, a sensitivity analysis was conducted using a fixed effects model in Revman [26] and the effect size was found to be similar. Thus, the findings of the analysis are robust.

### 4.3 Subgroup Analysis

Due to the paucity of data available for meta‐analysis, we did not conduct a subgroup analysis.

### 4.4 Effects of CBT—I on Pregnancy Mood

Most of the studies reported a positive impact of CBT‐I on maternal mood in pregnancy. Four out of five studies reporting on maternal mood found CBT‐to have a significant result in improving depression in the antenatal period [31, 33, 35, 36] with one other study reporting no change to post‐treatment depression scores [40] Two studies reported anxiety levels to be significantly reduced post the CBT‐I intervention [31, 33] with one study reporting no difference in the anxiety level [36]. Studies also mentioned a significant reduction in pregnancy stress scores [36] and a reduction in fatigue level [33] following the CBT‐I intervention.

### 4.5 Sleep Healthy Education

Two RCTs [34, 37] focused on specific sleep education during pregnancy. In both studies, the degree of sleep disturbance was measured through the PSQI score which decreased post intervention. Given, the limited studies and high statistical heterogeneity between them $I^{2}$ = 95%, meta‐analysis reporting was not possible. Sleep healthy training studied by Hassanpour et al. [34] reported the mean (Standard deviation) scores of the PSQI score for the intervention was 4.95 (2.05), and lower than the control group: 7.03 (0.78). This was statistically significant (*p <* 0.01). Similarly, Rezaei et al. [37] found a significant difference in PSQI mean score in the intervention group one‐month after the intervention compared to before the intervention (7.30 ± 3.33 vs. 8.76 ± 2.33, *p =* 0.009). Also, mean scores of the quality of life showed an increase at 1 month (*p <* 0.000) and 2 months (*p <* 0.001) after intervention in the study group compared to the control group.

One longitudinal pilot study [38] using the Sleep Health and Pregnancy Information (Sleep HAPi) education intervention reported the intervention group had a better measure of sleep quality which was statistically significant (*p =* 0.017), sleep continuity (*p* = 0.017) and sleep latency (*p =* 0.001) compared to the comparison group, but no significant differences were seen for sleep duration or daytime sleepiness. This study also evaluated the mood of the participants through the Edinburgh scoring and reported the participants in the intervention group experienced fewer depressive symptoms compared to the comparison group (*p =* 0.019), however, the anxiety subscale showed no difference between the groups (*p =* 0.18)

### 4.6 Relaxation Exercises

One study was found to study the impact of relaxation exercises training [39] during pregnancy to improve sleep. Post‐test PSQI mean scores were found to be lower in the relaxation exercises group than those in the control group (3.31 ± 1.96 vs. 8.74 ± 2.80; *p* = 0.001).

### 4.7 Potential Moderators

Studies including the CBT‐I intervention recruited pregnant women of varied gestation from as early as 13 weeks [29], up to 32 weeks [35]. Baseline characteristics of women varied between studies, and six studies targeted women with pre‐existing sleep disturbance [29, 31, 33, 34, 35, 37] and six healthy women without specific sleep disturbances [28, 30, 32, 36, 38, 39]. One study included obese or overweight pregnant women as its desired study population [29]. Controlling for the baseline characteristics of the women, the studies included in this review reported the psycho‐education intervention to be effective in reducing sleep disturbance.

The interventions were delivered in varied frequencies, with one study [34] offering the SHE in two 2‐h sessions that included a CD training package, to most studies delivering CBT‐I interventions in five to seven weekly sessions [29, 30, 31, 33, 35] (Table 2). The interventions were delivered through varied modalities, including individual face‐to‐face sessions [33, 35, 36, 37], digital interventions [30, 31, 32] and two studies offered the intervention in a group [29, 37]. Facilitators conducting the interventions varied between studies, such as psychologists [33] or trained therapists in psychology [35], an obstetrician and gynecologist [29] and digital animation therapists [30, 31]. Some studies did not clearly mention the provider of the intervention [34, 37, 38]. Despite the variation in the intervention delivery and the facilitators, none of the predicted moderators appeared to have negatively affected the measured sleep outcome and reports of improved sleep in pregnancy.

### 4.8 Acceptability of Intervention

Psycho‐educational interventions included in this review were generally well accepted by the women. Bei et al. [28] found participants were more satisfied with receiving CBT‐I (M ± S.D. = 81.17 ± 12.62) when compared to the control group who received only nutritional advice (74.14 ± 14.01) (*p =* 0.001). Qualitative data using open‐ended questions revealed women enjoyed the program and felt engaged [33]. In one study, women reported enjoying the group environment as it enabled sharing advice and ideas, and helped them to recognize they were not alone [33]. In most studies, attendance at sessions was good [28, 29, 30, 31, 32, 34, 35, 36, 39]. For instance, in the home‐based sleep enhancement training, most participants (89%–96%, or an average of 93%) completed the weekly readings, and most (78%–96%) would recommend the intervention to others [32]. Most studies used women‐reported outcome measures which were clinically related to their emotional well‐being and sleep quality [28, 29, 30, 31, 35]. In terms of challenges associated with CBT‐I participation, some women mentioned how they found it difficult to track their sleep [33], whilst participants in another study noted some challenges with accessing the reading materials and the audio programs [32]. Another study reported the session being conducted in a health center where there was not enough silence and comfort [34]. Two studies noted a limitation of their study was the majority of participants were highly educated, motivated women with stable jobs, which could question the generalizability of the findings to the other socio‐economic backgrounds [33, 39].

## 5 Discussion

To the best of our knowledge, this is the first integrative review that has aimed to critically appraise and determine if psychoeducational interventions optimize sleep in pregnancy, and to identify moderators in the treatment effects of the interventions under study. This review is different from previous studies in this field as it has included women in their pregnancy as compared to the postnatal period [17, 18]. Furthermore, this study assessed overall sleep quality as compared to maternal mental health outcomes (i.e., rate of stress, depression or anxiety) [18].

This review found that CBT‐I is effective in improving sleep in pregnant women. This finding is consistent with previous studies that found CBT‐I to be an effective intervention in treating chronic insomnia in non‐pregnant women [41, 42] and insomnia in pregnancy [20]. A few studies included in this review supported the efficacy and feasibility of SHE and relaxation training for improving sleep in pregnant women [34, 37, 38, 39]. Initial findings suggest these two interventions have the potential to improve sleep quality for pregnant women, and further research is required before making any recommendations.

CBT‐I is a safe, first‐line treatment for insomnia given it is more effective and acceptable than medications, and the effects may be more durable [42]. Studies testing different modes of CBT‐I delivery, including group CBT‐I therapy in the general population with insomnia [43] and e‐health intervention in pregnant women with diagnosed depression, anxiety, and insomnia [17] have found the intervention to be effective for improved sleep. However, in these studies, adherence to treatment was explained only in pregnant women from a Caucasian background, and hence further research is needed to explore adherence in women from diverse cultural backgrounds [33, 39]. An RCT conducted using digital CBT‐I reported treatment engagement to be lower in African American pregnant women compared to the white American women, resulting in poorer treatment outcomes in African American women [44]. As a result, recommendations to improve adherence to the sessions and treatment outcomes included more enhanced culturally tailored CBT‐I therapy with some modifications based on their specific social determinants of health [44]. Another RCT studying the efficacy of a culturally tailored version of the digital CBT‐I program found improved treatment adherence and treatment completion in African American women along with improved sleep quality compared to the standard digital sleep education [45]. These findings suggest culturally tailored digital CBT‐I may be an option for women who otherwise experience barriers to care.

Midwives are the primary contact for women in pregnancy and are therefore well placed to assess, screen, and discuss appropriate treatment options for psychological needs, including sleep disturbance, with women [46]. Research has found midwifery‐led psycho‐educational interventions, including CBT, to be effective in reducing the fear of childbirth [46], perinatal depression [47] and anxiety [48] as the pregnant women developed a safe and confiding relationship with their midwife and felt their mental health needs were listened to without judgment [49]. Training midwives in psychological treatments, such as CBT, has been supported by several studies that show this training helps in improving the perinatal mental health in women [47, 48, 49, 50] and could have a place in helping women with sleep disturbances in pregnancy. An Australian‐based review reported that midwives were interested in providing mental health support through psycho‐educational counseling interventions; however, midwives lacked the knowledge, confidence, and training to do so [49]. Appropriate psychological training, either as part of the continuing professional development or within the midwifery curriculum, is a feasible strategy to equip current and future midwives, and thereby the maternity workforce [47]. Expanding the scope of practice for midwives to incorporate psycho‐educational counseling interventions supports and promotes continuity of care, resulting in improved women‐centered care [49].

Sleep changes are very common in pregnancy, commencing early in the first trimester and gradually worsening over the course of pregnancy and the postnatal period [3, 51]. Research recommends an early intervention in pregnancy for sleep disturbances with appropriate follow‐up periods to avoid complications. Literature has shown CBT‐I to be an evidence‐based intervention for insomnia in pregnancy and is well received and accepted by women when delivered in a personally tailored manner [44, 45]. Midwives with training in psychological treatments such as CBT could provide this intervention in a timely manner and support women in improving sleep in pregnancy.

### 5.1 Limitations

This review has a number of limitations, primarily the availability of existing literature around the different psycho‐educational interventions. Related to this low availability, quantitative reporting was possible only for the CBT‐I interventions while further studies are needed to assess sleep education and relaxation training. Also, the review was unable to conduct sub‐analysis of the interventions based on their goal (treat or prevent) given the limited studies retrieved. Although some studies included a brief qualitative element to understand the women's acceptability of the intervention, there was no single qualitative study focusing more in‐depth on the perception of women about the psycho‐educational intervention to improve sleep in pregnancy. Also, two studies on CBT‐I included well educated, white women as their population which could limit the generalizability of the findings to diverse population. Success of the intervention could be provider dependent and could have some inconsistencies on the intervention's effectiveness. Thus, this is not a review of only high‐quality articles. However, the low‐quality articles were not included in the meta‐analysis of RCTs given the different study designs [33]. Due to the nature of the CBT‐I, blinding of the participants was not always possible by studies included in the metanalysis [29, 36] and some achieved partial blinding [30, 31, 35] which could potentially influence the findings. Only studies published in the English language were included, which could limit the results retrieved. Although CBT‐I sleep intervention has been associated with improved sleep quality, its relation to improved maternity outcomes needs further research.

## 6 Conclusions

Although psycho‐education seems to be a feasible and acceptable non‐invasive intervention for insomnia in pregnant women, there is currently insufficient evidence from high‐quality randomized trials on which to base the recommendations of the effectiveness of these interventions to improve sleep in pregnancy. Based on the available research comparing CBT‐I, SHE, and relaxation training, CBT‐I, regardless of the mode of delivery, was found to be an effective evidence‐based intervention to improve sleep quality in pregnancy. Midwives are the primary care providers for women in pregnancy, and with training in CBT‐I, they are well placed to provide these interventions with a woman‐centered focus. Future research into the other types of psycho‐educational sleep interventions, in‐depth exploration of women's perceptions of the interventions, and training for midwives on psychological interventions needs to be explored.

## Conflicts of Interest

The authors declare no conflicts of interest.

## Supporting information


Appendix S1.

Table S1.

Table S2.

## Data Availability

The authors have nothing to report.
