# Supplementary material for: The Efficacy of Psycho‐Educational Interventions to Optimize Women's Sleep in Pregnancy: An Integrative Review
Source: Birth. 2025 Mar 10;52(2):228–42. doi: 10.1111/birt.12902 (PMC12060623; doi:10.1111/birt.12902)
Supplement: Supplementary file 1 — Appendix S1. [file BIRT-52-228-s001.docx]

**Appendix S1:** Search Strategy

Final search conducted on: 20^th^ December 2022

*CINAHL Search strategy*

| **#** | **Query** | **Limiters/Expanders** | **Last Run Via** |  | **Results** |
| --- | --- | --- | --- | --- | --- |
| S28 | S8 AND S18 AND S26 | Limiters - Published  Date: 19760101-  20231231  Search modes Boolean/Phrase | Interface - EBSCOhost  Research Databases  Search Screen - Advanced  Search  Database - CINAHL Complete |  | 251 |
| S27 | S8 AND S18 AND S26 | Search modes -  Boolean/Phrase | Interface - EBSCOhost  Research Databases  Search Screen - Advanced  Search  Database - CINAHL Complete |  | 251 |
| S26 | S19 OR S20 OR S21 OR  S22 OR S23 OR S24 OR  S25 | Search modes -  Boolean/Phrase | Interface - EBSCOhost  Research Databases  Search Screen - Advanced  Search  Database - CINAHL Complete |  | 102,215 |
| S25 | TI "insomnia" OR AB  "insomnia" | Search modes -  Boolean/Phrase | Interface - EBSCOhost  Research Databases  Search Screen - Advanced  Search  Database - CINAHL Complete |  | 11,206 |
| S24 | TI "sleep apn?a" OR AB  "sleep apn?a" | Search modes -  Boolean/Phrase | Interface - EBSCOhost  Research Databases  Search Screen - Advanced  Search  Database - CINAHL Complete |  | 13,282 |
| S23 | TI "sleep*" OR AB  "sleep*" | Search modes -  Boolean/Phrase | Interface - EBSCOhost  Research Databases  Search Screen – Advanced  Search  Database - CINAHL Complete |  | 80,079 |
| S22 | TI "sleep complaint*" OR  AB "sleep complaint*" | Search modes -  Boolean/Phrase | Interface - EBSCOhost  Research Databases  Search Screen - Advanced  Search  Database - CINAHL Complete |  | 515 |

| S21 | TI "sleep disorder*" OR  AB "sleep disorder*" | Search modes -  Boolean/Phrase | Interface - EBSCOhost  Research Databases  Search Screen - Advanced  Search  Database - CINAHL Complete | 8,308 |
| --- | --- | --- | --- | --- |
| S20 | TI "sleep qualit*" OR AB  "sleep qualit*" | Search modes -  Boolean/Phrase | Interface - EBSCOhost  Research Databases  Search Screen - Advanced  Search  Database - CINAHL Complete | 10,364 |
| S19 | (MH "Sleep+") OR (MH  "Sleep Apnea, Central+")  OR (MH "Sleep-Wake  Transition Disorders+")  OR (MH "Sleep  Disorders, Intrinsic+") OR  (MH "Sleep Apnea  Syndromes+") OR (MH  "Sleep Arousal  Disorders+") OR (MH  "Sleep Disorders+") OR  (MH "Sleep Latency") OR  (MH "Sleep Quality") OR  (MH "Sleep Pattern  Disturbance (Saba  CCC)") OR (MH "Sleep  Pattern Control (Saba  CCC)") OR (MH "Sleep  Deprivation (Saba  CCC)") OR (MH "Sleep  Enhancement (Iowa  NIC)") OR (MH "Sleep  Deprivation") OR (MH  "Insomnia+") | Search modes -  Boolean/Phrase | Interface - EBSCOhost  Research Databases  Search Screen - Advanced  Search  Database - CINAHL Complete | 66,038 |
| S18 | S9 OR S10 OR S11 OR  S12 OR S13 OR S14 OR  S15 OR S16 OR S17 | Search modes -  Boolean/Phrase | Interface - EBSCOhost  Research Databases  Search Screen - Advanced  Search  Database - CINAHL Complete | 212,732 |
| S17 | TI "psycho-education" OR  AB "psycho-education" | Search modes -  Boolean/Phrase | Interface - EBSCOhost  Research Databases  Search Screen - Advanced  Search  Database - CINAHL Complete | 2,243 |

| S16 | TI "sleep health" OR AB  "sleep health" | Search modes -  Boolean/Phrase | Interface - EBSCOhost  Research Databases  Search Screen - Advanced  Search  Database - CINAHL Complete | 538 |
| --- | --- | --- | --- | --- |
| S15 | TI "sleep hygiene" OR AB  "sleep hygiene" | Search modes -  Boolean/Phrase | Interface - EBSCOhost  Research Databases  Search Screen - Advanced  Search  Database - CINAHL Complete | 1,044 |
| S14 | "cognitive behavio*" OR  "cognitive behavio*" | Search modes -  Boolean/Phrase | Interface - EBSCOhost  Research Databases  Search Screen - Advanced  Search  Database - CINAHL Complete | 18,000 |
| S13 | TI "CBT" OR AB "CBT" | Search modes -  Boolean/Phrase | Interface - EBSCOhost  Research Databases  Search Screen - Advanced  Search  Database - CINAHL Complete | 6,984 |
| S12 | (MH "Sleep Hygiene+")  OR (MH "Sleep  Enhancement (Iowa  NIC)") | Search modes -  Boolean/Phrase | Interface - EBSCOhost  Research Databases  Search Screen - Advanced  Search  Database - CINAHL Complete | 2,109 |
| S11 | (MH "Cognitive  Therapy+") OR (MH  "Behavior Therapy+") OR  (MH "Cognitive  Restructuring") OR (MH  "Cognitive Therapy (Iowa  NIC)+") OR (MH  "Behavior Therapy (Iowa  NIC)+") OR (MH  "Behavioral Changes") OR (MH "Behavior  Modification") OR (MH  "Maternal Behavior") | Search modes -  Boolean/Phrase | Interface - EBSCOhost  Research Databases  Search Screen - Advanced  Search  Database - CINAHL Complete | 59,418 |
| S10 | (MH "Patient  Education+") OR (MH  "Patient Education (Iowa NIC)+") OR (MH "Health  Education+") OR (MH | Search modes -  Boolean/Phrase | Interface - EBSCOhost  Research Databases  Search Screen - Advanced  Search  Database - CINAHL Complete | 142,565 |

|  | "Parent Education:  Childrearing Family (Iowa  NIC)") OR (MH "Parent  Education: Childbearing  Family (Iowa NIC)") OR (MH "Parent Education:  Adolescent (Iowa NIC)")  OR (MH "Online  Education") |  |  |  |
| --- | --- | --- | --- | --- |
| S9 | (MH "Psycho-education") | Search modes -  Boolean/Phrase | Interface - EBSCOhost  Research Databases  Search Screen - Advanced  Search  Database - CINAHL Complete | 3,925 |
| S8 | S1 OR S2 OR S3 OR S4  OR S5 OR S6 OR S7 | Search modes -  Boolean/Phrase | Interface - EBSCOhost  Research Databases  Search Screen - Advanced  Search  Database - CINAHL Complete | 280,249 |
| S7 | TI "antepartum" OR AB  "antepartum" | Search modes -  Boolean/Phrase | Interface - EBSCOhost  Research Databases  Search Screen - Advanced  Search  Database - CINAHL Complete | 2,389 |
| S6 | TI "antenatal" OR AB  "antenatal" | Search modes -  Boolean/Phrase | Interface - EBSCOhost  Research Databases  Search Screen - Advanced  Search  Database - CINAHL Complete | 18,136 |
| S5 | TI "prenatal" OR AB  "prenatal" | Search modes -  Boolean/Phrase | Interface - EBSCOhost  Research Databases  Search Screen - Advanced  Search  Database - CINAHL Complete | 32,445 |
| S4 | TI "pregnan#" OR AB  "pregnan#" | Search modes -  Boolean/Phrase | Interface - EBSCOhost  Research Databases  Search Screen - Advanced Search  Database - CINAHL Complete | 62,050 |
| S3 | (MH "Prenatal Care") OR  (MH "Prenatal  Diagnosis") OR (MH “Prenatal Care(lowa NIC)” | Search modes -  Boolean/Phrase | Interface - EBSCOhost  Research Databases  Search Screen - Advanced Search  Search  Database - CINAHL Complete | 29,306 |

| S2 (MH "Pregnancy+") OR Search modes - Interface - EBSCOhost 245,145  (MH "Pregnancy in Boolean/Phrase Research Databases  Adolescence+") OR (MH Search Screen - Advanced  "Pregnancy Trimesters+") Search  OR (MH "Pregnancy, Database - CINAHL Complete  Unplanned") OR (MH  "Pregnancy,  Heterotopic") OR (MH  "Pregnancy Outcomes")  OR (MH "Pregnancy  Discomforts") OR (MH  "Pregnancy, Quadruplet")  OR (MH "Pregnancy,  Unwanted") OR (MH  "Pregnancy, Twin") OR  (MH "Pregnancy, Triplet")  OR (MH "Pregnancy,  Quintuplet") OR (MH  "Pregnancy, Prolonged")  OR (MH "Pregnancy,  Multiple+") OR (MH  "Pregnancy, High Risk")  OR (MH "Pregnancy  Trimester, Third") OR  (MH "Pregnancy  Trimester, Second") OR  (MH "Pregnancy  Trimester, First")  S1 (MH "Expectant Search modes - Interface - EBSCOhost 12,919 Mothers") Boolean/Phrase Research Databases  Search Screen - Advanced  Search  Database - CINAHL Complete |
| --- |

*SCOPUS Search strategy*

306 results matching ( ( TITLE-ABS-KEY ( *"sleep"* ) )  OR  ( TITLE-ABS-KEY ( *"sleep qualit*"* ) )  OR  ( TITLE-ABS-KEY ( *"sleep complaint*"* ) )  OR  ( TITLE-ABS-KEY ( *"sleep disorder*"* ) )  OR  ( TITLE-ABS-KEY ( *"sleep apnea"* ) ) )  AND  ( ( TITLE-ABS-KEY ( *"*psycho-education*"* ) )  OR  ( TITLE-ABS-KEY ( *"sleep hygiene"* ) )  OR  ( TITLE-ABS-KEY ( *"sleep education"* ) )  OR  ( TITLE-ABS-KEY ( *"cognitive behavio*"* ) )  OR  ( TITLE-ABS-KEY ( *"CBT"* ) )  OR  ( TITLE-ABS-KEY ( *"Sleep health"* ) ) )  AND  ( ( TITLE-ABS-KEY ( *"pregnant wom?n"* ) )  OR  ( TITLE-ABS-KEY ( *"pregnan*"* ) )  OR  ( TITLE-ABS-KEY ( *"prenatal"* ) )  OR  ( TITLE-ABS-KEY ( *"antenatal"* ) )  OR  ( TITLE-ABS-KEY ( *"antepartum"* ) ) )

*OVID Medline Search strategy*

|  | **Searches** | **Results** |
| --- | --- | --- |
| 1 | exp Pregnant Women/ | 14191 |
| 2 | exp Pregnancy, Triplet/ or exp Pregnancy Outcome/ or exp Pregnancy, Quintuplet/ or exp Pregnancy Trimester, First/ or exp Pregnancy, Multiple/ or exp Pregnancy, Prolonged/ or exp Pregnancy/ or exp Pregnancy, High-Risk/ or exp Pregnancy Trimester, Second/ or exp Pregnancy, Unwanted/ or exp Pregnancy, Twin/ or exp Pregnancy, Quadruplet/ or exp Pregnancy, Unplanned/ or exp Pregnancy, Heterotopic/ or exp Pregnancy in Adolescence/ or exp Pregnancy Trimester, Third/ or exp Pregnancy Complications/ | 1034873 |
| 3 | exp Prenatal Care/ or exp Prenatal Diagnosis/ | 111284 |
| 4 | pregnan*.ti,ab. | 529685 |
| 5 | prenatal.ti,ab. | 99909 |
| 6 | antenatal.ti,ab. | 38059 |
| 7 | antepartum.ti,ab. | 6037 |
| 8 | 1 or 2 or 3 or 4 or 5 or 6 or 7 | 1135452 |
| 9 | exp Patient Education as Topic/ or exp Psychotherapy/ | 301961 |
| 10 | exp Health Education/ed [Education] | 72 |
| 11 | exp Cognitive Behavioral Therapy/ | 36267 |
| 12 | exp Sleep Hygiene/ | 1912 |
| 13 | cbt.ti,ab. | 11565 |
| 14 | Cognitive Behavio*.ti,ab. | 29067 |
| 15 | sleep hygiene.ti,ab. | 1623 |
| 16 | sleep health.ti,ab. | 1051 |
| 17 | psycho-education.ti,ab. | 3353 |
| 18 | 9 or 10 or 11 or 12 or 13 or 14 or 15 or 16 or 17 | 318284 |
| 19 | exp Sleep Apnea, Obstructive/ or exp "Sleep Initiation and Maintenance Disorders"/ or exp Sleep Quality/ or exp Sleep Apnea, Central/ or exp Sleep Arousal Disorders/ or exp Sleep-Wake Transition Disorders/ or exp Sleep Latency/ or exp Sleep/ or exp Sleep Apnea Syndromes/ or exp Sleep Disorders, Intrinsic/ or exp Sleep Wake Disorders/ or exp Sleep Deprivation/ | 170674 |
| 20 | sleep.ti,ab. | 179658 |
| 21 | sleep qualit*.ti,ab. | 18572 |
| 22 | sleep complaint*.ti,ab. | 1198 |
| 23 | sleep disorder*.ti,ab. | 20598 |
| 24 | sleep apn?a.ti,ab. | 32935 |
| 25 | insomnia.ti,ab. | 23234 |
| 26 | 19 or 20 or 21 or 22 or 23 or 24 or 25 | 227573 |
| 27 | 8 and 18 and 26 | 314 |
| 28 | limit 27 to yr="1976 - 2023" | 298 |

## *OVID Embase Search strategy*

|  | **Searches** | **Results** |
| --- | --- | --- |
| 1 | exp Pregnant Women/ | 14191 |
|  |  |  |
| 2 | exp Pregnancy, Triplet/ or exp Pregnancy Outcome/ or exp Pregnancy, Quintuplet/ or exp Pregnancy Trimester, First/ or exp Pregnancy, Multiple/ or exp Pregnancy, Prolonged/ or exp Pregnancy/ or exp Pregnancy, High-Risk/ or exp Pregnancy Trimester, Second/ or exp Pregnancy, Unwanted/ or exp Pregnancy, Twin/ or exp Pregnancy, Quadruplet/ or exp Pregnancy, Unplanned/ or exp Pregnancy, Heterotopic/ or exp Pregnancy in Adolescence/ or exp Pregnancy Trimester, Third/ or exp Pregnancy Complications/ | 1034873 |
| 3 | exp Prenatal Care/ or exp Prenatal Diagnosis/ | 111284 |
| 4 | pregnan*.ti,ab. | 529685 |
| 5 | prenatal.ti,ab. | 99909 |
| 6 | antenatal.ti,ab. | 38059 |
| 7 | antepartum.ti,ab. | 6037 |
| 8 | 1 or 2 or 3 or 4 or 5 or 6 or 7 | 1135452 |
| 9 | exp Patient Education as Topic/ or exp Psychotherapy/ | 301961 |
| 10 | exp Health Education/ed [Education] | 72 |
| 11 | exp Cognitive Behavioral Therapy/ | 36267 |
| 12 | exp Sleep Hygiene/ | 1912 |
| 13 | cbt.ti,ab. | 11565 |
| 14 | Cognitive Behavio*.ti,ab. | 29067 |
| 15 | sleep hygiene.ti,ab. | 1623 |
| 16 | sleep health.ti,ab. | 1051 |
| 17 | psycho-education.ti,ab. | 3353 |
| 18 | 9 or 10 or 11 or 12 or 13 or 14 or 15 or 16 or 17 | 318284 |
| 19 | exp Sleep Apnea, Obstructive/ or exp "Sleep Initiation and Maintenance Disorders"/ or exp Sleep Quality/ or exp Sleep Apnea, Central/ or exp Sleep Arousal Disorders/ or exp Sleep-Wake Transition Disorders/ or exp Sleep Latency/ or exp Sleep/ or exp Sleep Apnea Syndromes/ or exp Sleep Disorders, Intrinsic/ or exp Sleep Wake Disorders/ or exp Sleep Deprivation/ | 170674 |
| 20 | sleep.ti,ab. | 179658 |
| 21 | sleep qualit*.ti,ab. | 18572 |
| 22 | sleep complaint*.ti,ab. | 1198 |
| 23 | sleep disorder*.ti,ab. | 20598 |
| 24 | sleep apn?a.ti,ab. | 32935 |
| 25 | insomnia.ti,ab. | 23234 |
| 26 | 19 or 20 or 21 or 22 or 23 or 24 or 25 | 227573 |
| 27 | 8 and 18 and 26 | 314 |
| 28 | limit 27 to yr="1976 - 2023" | 298 |

## *OVID Psycinfo Search strategy*

|  | **Searches** | **Results** |
| --- | --- | --- |
| 1 | exp Pregnancy/ | 48318 |
| 2 | exp Prenatal Care/ or exp Prenatal Diagnosis/ | 3216 |
| 3 | exp Antepartum Period/ | 447 |
| 4 | pregnan*.ti,ab. | 53551 |
| 5 | prenatal.ti,ab. | 20901 |
| 6 | antenatal.ti,ab. | 4220 |
| 7 | antepartum.ti,ab. | 390 |
| 8 | 1 or 2 or 3 or 4 or 5 or 6 or 7 | 83671 |
| 9 | exp Psycho-education/ | 5408 |
| 10 | exp Cognitive Behavior Therapy/ or exp Group Psychotherapy/ or exp Cognitive Therapy/ | 61852 |
| 11 | exp Client Education/ | 4576 |
| 12 | cbt.ti,ab. | 16573 |
| 13 | cognitive behavio*.ti,ab. | 47046 |
| 14 | sleep hygiene.ti,ab. | 1116 |
| 15 | Sleep health.ti,ab. | 559 |
| 16 | psycho-education.ti,ab. | 5302 |
| 17 | 9 or 10 or 11 or 12 or 13 or 14 or 15 or 16 | 94441 |
| 18 | exp Sleep Quality/ or exp Sleep Apnea/ or exp Sleep Treatment/ or exp Sleep Wake Disorders/ or exp Sleep/ or exp Sleep Wake Cycle/ or exp Sleep Deprivation/ or exp Sleep Onset/ | 63435 |
| 19 | exp Insomnia/ | 8115 |
| 20 | sleep.ti,ab. | 79177 |
| 21 | sleep qualit*.ti,ab. | 9646 |
| 22 | sleep complaint*.ti,ab. | 825 |
| 23 | sleep disorder*.ti,ab. | 8279 |
| 24 | sleep apn?a.ti,ab. | 4482 |
| 25 | insomnia.ti,ab. | 14567 |
| 26 | 18 or 19 or 20 or 21 or 22 or 23 or 24 or 25 | 94491 |
| 27 | 8 and 17 and 26 | 78 |
| 28 | limit 27 to yr="1976 - 2023" | 78 |
