# Supplementary material for: The Efficacy of Psycho‐Educational Interventions to Optimize Women's Sleep in Pregnancy: An Integrative Review
Source: Birth. 2025 Mar 10;52(2):228–42. doi: 10.1111/birt.12902 (PMC12060623; doi:10.1111/birt.12902)
Supplement: Supplementary file 2 — Table S1. [file BIRT-52-228-s002.docx]

**Table S1: Quality Assessment**

**Quality assessment using the “QualSyst” Tool**

| Study ID | Question / objective sufficiently described? | Study design evident and appropriate? | Method of subject/comparison group selection or source of information/input variables described and appropriate? | Subject (and comparison group, if applicable) characteristics sufficiently described? | If interventional and random allocation was possible, was it described? | If interventional and blinding of investigators was possible, was it reported? | If interventional and blinding of subjects was possible, was it reported? | Outcome and (if applicable) exposure measures well defined and robust to measurement / misclassification bias? Means of assessment reported? | Sample size appropriate? | Analytical methods described / justified and appropriate? | Some estimate of variance is reported for main results? | Controlled for confounding? | Results reported in sufficient detail? | Conclusions supported by results? | Summary score and Quality of article |
| --- | --- | --- | --- | --- | --- | --- | --- | --- | --- | --- | --- | --- | --- | --- | --- |
| Bei 2021 | Yes (2) | Yes (2) | Yes (2) | Yes (2) | Yes (2) | No (0) | Yes (2) | Yes (2) | Yes (2) | Yes (2) | Yes (2) | Yes (2) | Yes (2) | Yes (2) | Strong (Summary score >0.80) |
| Cain 2020 | Yes (2) | Yes (2) | Yes (2) | Yes (2) | Yes (2) | No (0) | No (0) | Partial (1) | Partial (1) | Yes (2) | Yes (2) | Partial (1) | Yes (2) | Yes (2) | good (score: 0.71-0.79) |
| Felder 2020 | Yes (2) | Yes (2) | Yes (2) | Yes (2) | Yes (2) | Partial (1) | Partial (1) | Partial (1) | Yes (2) | Yes (2) | Yes (2) | Partial (1) | Yes (2) | Yes (2) | Strong (Summary score >0.80) |
| Hassanpour 2014 | Yes (2) | Yes (2) | Yes (2) | Partial (1) | Yes (2) | No (0) | No (0) | Partial (1) | Yes (2) | Yes (2) | Yes (2) | Partial (1) | Yes (2) | Partial (1) | good (score: 0.71-0.79) |
| Kalmbach 2020 | Yes (2) | Yes (2) | Partial (1) | Yes (2) | Yes (2) | Partial (1) | Partial (1) | Yes (2) | Yes (2) | Yes (2) | Yes (2) | Partial (1) | Partial (1) | Yes (2) | good (score: 0.71-0.79) |
| Khatibi 2021 | Yes (2) | Yes (2) | Yes (2) | Yes (2) | Yes (2) | No (0) | No (0) | Partial (1) | Yes (2) | Yes (2) | Yes (2) | Partial (1) | Yes (2) | Yes (2) | good (score: 0.71-0.79) |
| Ladyman 2020 | Yes (2) | Yes (2) | Partial (1) | Yes (2) | NA | No (0) | No (0) | Partial (1) | No (0) | Yes (2) | Yes (2) | Partial (1) | Yes (2) | Partial (1) | adequate (score- 0.50-0.70) |
| Lee 2016 | Yes (2) | Yes (2) | Partial (1) | Partial (1) | NA | No (0) | NA | Partial (1) | Yes (2) | Yes (2) | Yes (2) | Partial (1) | Yes (2) | Partial (1) | adequate (score- 0.50-0.70) |
| Manber 2019 | Yes (2) | Yes (2) | Yes (2) | Yes (2) | Yes (2) | Yes (2) | Partial (1) | Yes (2) | Yes (2) | Yes (2) | Yes (2) | Yes (2) | Yes (2) | Yes (2) | Strong (Summary score >0.80) |
| Ozkan 2018 | Yes (2) | Yes (2) | Yes (2) | Yes (2) | Yes (2) | No (0) | No (0) | Partial (1) | Yes (2) | Yes (2) | Yes (2) | Yes (2) | Yes (2) | Yes (2) | Strong (Summary score >0.80) |
| Rezaei 2014 | Partial (1) | Yes (2) | Yes (2) | Yes (2) | Yes (2) | No (0) | Yes (2) | Partial (1) | Partial (1) | Partial (1) | Yes (2) | Partial (1) | Partial (1) | Partial (1) | adequate (score- 0.50-0.70) |
| Tomfohr-Madsen 2017 | Partial (1) | Partial (1) | Yes (2) | Partial (1) | NA | No (0) | No (0) | Partial (1) | No (0) | Partial (1) | Partial (1) | Partial (1) | Partial (1) | Partial (1) | Limited (Score <0.50) |
