# Supplementary material for: The Efficacy of Psycho‐Educational Interventions to Optimize Women's Sleep in Pregnancy: An Integrative Review
Source: Birth. 2025 Mar 10;52(2):228–42. doi: 10.1111/birt.12902 (PMC12060623; doi:10.1111/birt.12902)
Supplement: Supplementary file 3 — Table S2. [file BIRT-52-228-s003.docx]

| Study | Primary outcomes | Secondary outcome |
| --- | --- | --- |
| Bei 2021 | Insomnia severity & Sleep disturbance: ISI & PROMIS | SDM- 5 Insomnia disorder, Sleep diary, PROMIS short forms to measure sleep impairment, depression and anxiety |
| Cain 2020 | PSQI, ISI |  |
| Felder 2020 | ISI, Sleep diary, PSQI | GAD, EPDS |
| Hassanpour 2014 | PSQI |  |
| Kalmbach 2020 | The ISI, PSQI, EPDS, Pre-Sleep Arousal Scale's Cognitive factor (PSAS-C) |  |
| Khatibi 2021 | Sleep quality: PSQI | Depression, Anxiety and Stress : DASS |
| Ladyman 2020 | Sleep characteristics using GSDS & EPDS |  |
| Lee 2016 | Sleep duration and sleep disturbance using wrist actigraphy |  |
| Manber 2019 | Insomnia Severity: ISI | Self- reported awake time, Insomnia remission period, EPDS |
| Ozkan 2018 | PSQI |  |
| Rezaei 2014 | Sleep quality measure PSQI  Quality of life WHOQOL |  |
| Tomfohr-Madsen 2017 | Sleep measurement: ISI, PSQI, Actigraphy, Sleep diary | EPDS, Pregnancy specific anxiety measure, Multidimensional Fatigue Symptom Inventory–Short Form (MFSI-SF) |

**Table S2: Study outcomes**
